# Client satisfaction with antiretroviral treatment services in South Ethiopian public health facilities: an institution-based cross-sectional survey

**DOI:** 10.1080/16549716.2023.2212949

**Published:** 2023-05-17

**Authors:** Abebe Sorsa Badacho, Abera Chama, Tadele Dana Darebo, Deginesh Dawit Woltamo

**Affiliations:** a Department of Health Services Management, School of Public Health, College of Health Sciences and Medicine, Wolaita Sodo University Ethiopia, Wolaita Sodo, Ethiopia; b Department of Reproductive Health and Nutrition, School of Public Health, College of Health Sciences and Medicine, Wolaita Sodo University Ethiopia, Wolaita Sodo, Ethiopia; c Department of Epidemiology and Biostatistics, School of Public Health, College of Health Sciences and Medicine, Wolaita Sodo University Ethiopia, Wolaita Sodo, Ethiopia

**Keywords:** Client satisfaction, antiretroviral treatment services, HIV, AIDS, public health facilities

## Abstract

**Background:**

HIV/AIDS remains the leading cause of morbidity and mortality worldwide. Moreover, sub-Saharan countries, including Ethiopia, are highly affected by HIV/AIDS pandemic. Ethiopia’s government has been working on a comprehensive HIV care and treatment programme, including antiretroviral therapy. However, evaluating client satisfaction with antiretroviral treatment services is not well studied.

**Objectives:**

This study aimed to assess client satisfaction and associated factors with antiretroviral treatment services provided at public health facilities of Wolaita zone, South Ethiopia.

**Methods:**

A facility-based cross-sectional study involved 605 randomly selected clients using ART services from six public health facilities in Southern Ethiopia. A multivariate regression model was used to see an association between independent variables and the outcome variable. The odds ratio with 95% CI was computed to determine the presence and strength of the association.

**Results:**

Four hundred twenty-eight (70.7%) clients were satisfied with an overall antiretroviral treatment service, which included significant variations ranging from 21.1% to 90.0% among health facilities. Sex [AOR = 1.91; 95% CI = 1.10–3.29], employment [AOR = 13.04; 95% CI = 4.34–39.22], clients’ perception of the availability of prescribed laboratory services [AOR = 2.56; 95% CI = 1.42–4.63], availability of prescribed drugs [AOR = 6.26; 95% CI = 3.40–11.52] and cleanliness of toilet in the facility [AOR = 2.83; 95% CI = 1.56–5.14] were factors associated with client satisfaction with antiretroviral treatment services.

**Conclusion:**

The overall client satisfaction with antiretroviral treatment service was lower than the national target of 85%, with a marked difference among facilities. Sex, occupational status, availability of comprehensive laboratory services, standard drugs, and cleanliness toilets in the facility were factors associated with client satisfaction with antiretroviral treatment services. Sex-sensitive services needed to address and sustained availability of laboratory services and medicine recommended.

## Introduction

Antiretroviral treatment (ART) has been influential in managing HIV/AIDS since its discovery [1]. The public health sector provides HIV care and treatment services for people living with HIV (PLHIV) throughout ART centres. The ART centres provide comprehensive services to all PLHIV clients enrolled on the programme. Initial clinical evaluation, counselling, provision of ARV (antiretroviral) drugs, prophylaxis and management of opportunistic infections, and regular follow-up of patients are services included under the programme. Globally, highly active antiretroviral treatment (HAART) provisions have shown significant progress by decreasing HIV/AIDS-related mortality and new incidence by 45% and 23% in the last decades [2].

In Ethiopia, free ARV service was launched in January 2005 and started providing free ARV services in March 2005 in public hospitals and health centres [2,3].

Client satisfaction is a commonly used outcome measure of patient care and an essential indicator of health care performance services [3,4]. Clients have explicit requests for different services when they visit health facilities. However, many reasons for clients’ dissatisfaction can occur due to the unavailability of their needs or services [5]. Client satisfaction occurs when clients feel their needs and expectations are being met by the service delivery they receive in health institutions, measured by the client’s perception of care received compared with the care expected [6–8]. Client satisfaction is a vital component to improving services’ efficiency; eliciting the opinion of users about the available services and its determinant factors associated with dissatisfaction is critical [6–8].

Patients’ or clients’ satisfaction data are routinely collected and used for continuous quality improvement by healthcare institutions, including hospitals and health centres in developing countries [9]. It is measured over various health service dimensions, including availability, accessibility and convenience, the provider’s technical competence, interpersonal skills, and the physical environment where services are delivered [3,8]. Patient satisfaction is a ‘patient-centred’ process measure, reflecting the patients’ responses to and evaluation of care [10].

Some medical care and service satisfaction studies showed that clients with poor health status had stronger feelings about all healthcare services. Significantly, the most satisfied groups in service satisfaction were those with good health status [5–7,11,12]. However, client satisfaction was not only dependent on the client’s health. Poor immune and virological responses were also significantly associated with client satisfaction [7,13,14]. Very high levels of adherence (>95%) are necessary for sustained clinical success as well as ART service satisfaction [15].

In Ethiopia, patients or clients satisfaction studies suggest that factors are related to the quality of services given in health institutions [16,17]. According to a study conducted in Amhara region hospitals, laboratory services, waiting hours during registration, visitation of doctors after registration, acquisition of drugs from the hospitals’ pharmacies, and re-visiting of the doctor/health professionals for evaluation with laboratory results were identified factors associated with client dissatisfaction [16,17].

Several associated factors that influence patients’ satisfaction with health care services include patients’ socio-demographic characteristics, physical health Status, patients’ understanding and expectations from various healthcare services, and doctors, nurses, laboratory, and pharmacy services [18]. Poor patient satisfaction is associated with inadequate virological and immunological responses to the disease. It is also responsible for developing resistant strains and the death of patients.

Waiting time, absence of care provider on time, lack of laboratory service, access and cleanliness of the health latrines affect client satisfaction [10].

Some studies investigated client satisfaction with ART services and their associated factors in Ethiopia [3,6,10,17]. Most of these studies focus on service provision to ART clinics & some studies are conducted on client satisfaction in the specific department (laboratory services). However, limited studies were conducted on client satisfaction with ART services and its associated factors in the study area.

**Figure 1.**
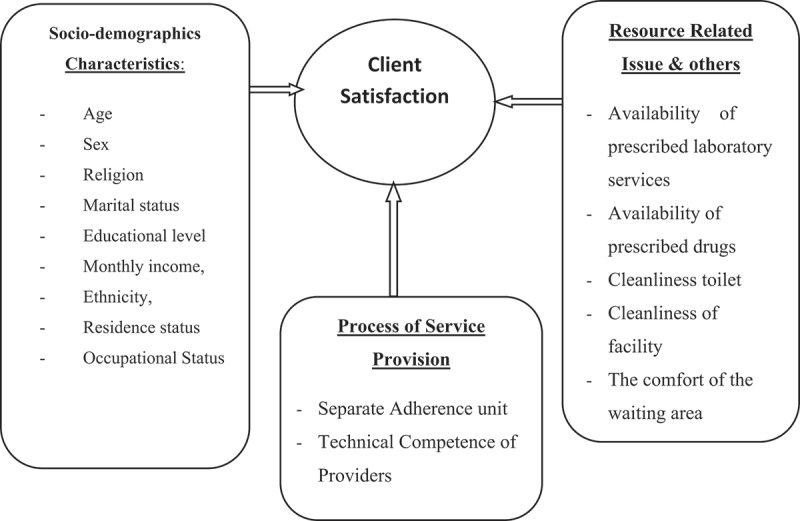
Source of conceptual framework adapted from other works of literature.

Therefore, this study aimed to assess client satisfaction with ART services and identify predictors of client satisfaction with ART services (Figure 1).

## Methods

### Study design

A facility-based cross-sectional study design was used in August and September 2021.

### Study setting

A study was conducted in the Wolaita zone of south Ethiopia with an estimated total population of 2,067,163, of whom 50.46% are females. The zone has one teaching referral hospital, two private general hospitals, four primary government hospitals, 68 health centres, and 358 health posts. Seven hospitals and eleven public health centres have provided ART services for 4231 ART clients. ART clients aged 18 years and above attending ART clinics for at least six months were included in the study.

#### Study population

All ART clients active in care at participating public health facilities in the Wolaita zone comprised the study population.

#### Sample size and sampling procedures

The single population proportion formula was used to calculate the sample size: *n* = (Z/2)2 p (1-p)DE/d2. Considering the prevalence of the Hossana study (70.1% client satisfaction rate) [6]. Marginal error (d) of 5%, confidence interval of 95%, and Zα/2 is the value of the standard average distribution corresponding to a significant level of alpha (α) 0.05 and 2 is the design effect.

$$n=((Za/2{)}^{2}p(1-p))2/d^{2},$$

where: *n* = the required sample size, Zα/2 = 95% confidence level of certainty (1.96) at critical value, DE = design effect (2), d = is the margin of error between the sample (n) and the population (N) or desired precision 5% (0.05) & P = prevalence of Client Satisfaction Level 70.1% (0.701) with the total sample size was 615 clients from each selected ART center in the facility.

#### Sampling techniques

Six public health facilities with ART clinics providing HIV care for people living with HIV were randomly selected, and six hundred fifteen study participants were selected using a random sampling technique. The total number of study participants for each ART clinic was calculated using the proportional allocation for six public health facilities providing ART services. Adults aged 18 years and above who were receiving ART were included in the study. Individuals asked for face-to-face interviews when they came for ART services. The total sample size estimated was 615, and 605 responded to the interview; from the contacted persons for the interview, ten were not responded, giving a response rate of 98.4%.

### Measurements

#### Client satisfaction

Client satisfaction was measured using fourteen Likert scale items, ranging from ‘strongly disagree to ‘strongly agree’, with the sum of a minimum satisfaction score of 14 and a maximum satisfaction score of 70 on the service provision process. Each response was categorised and scored as follows: ‘strongly disagree’ = 1, ‘disagree’ = 2, ‘uncertain’ = 3, ‘agree’ = 4, and ‘strongly agree’ = 5. Furthermore, each study participant’s sum of each score was converted to a percentage score (computed using SPSS), and the overall satisfaction was considered the sum of a percent satisfaction score of 75% and above as satisfied.

### Data collection

#### Data collection instrument

The data collection tool was an interviewer-administered structured questionnaire (demographic variables, client’s perception of the availability of resources and service delivery). The English version was translated into the local language (Amharic) used for gathering information and again translated to English to confirm the correctness of the translation by language expert.

#### Quality control

The data quality was done by checking data accuracy and outliers and handling missed values. Training of data collectors and properly designed pretests was established before the data collection. Four diploma and two BSC nurses were recruited and trained as data collectors and supervisors. The training consisted of the study objective, the introduction of the questionnaire and the procedure of interviewing the study participant. Pretesting was done in 5% of the total sample size and was not included in the study.

#### Data processing and analysis

Double verification was done for data entry and cleaning using the statistical programme EPI Info 3.5.3. The SPSS version 21 software programme was used to conduct the analysis. The dependent variable was client satisfaction with ART services. The binary logistic regression model performed bivariate analysis, and *p*-value cut-off points less than 0.25 were chosen as the candidate variable for multivariate analysis. Using the odds ratio, statistical tests were conducted between dependent and independent factors. Age, sex, level of education, marital status, occupation, place of residence, availability to prescribed laboratory services, availability to prescribed medications, cleanliness of the facility’s toilets, the facility’s cleanliness and the service providers’ technical ability were potential candidate variables for multivariate analysis. An adjusted odds ratio with a 95% confidence interval and a P-value of 0.05 was used to determine statistical significance. Before performing multivariable analysis, multicollinearity was examined. The final model was examined for goodness-of-fit using the Hosmer–Lemeshow test (*p* > 0.05). Texts and tables were used to present the findings.

## Results

### Socio-demographic characteristics of study participants

Six hundred and five study participants participated in the study, yielding a 98.4% response rate. More than half (56.9%) of the participants were female. About a third, 182 (30.1%) of the participants were between 18 and 30 years of age, and more than half, 327 (54.0%) participants were between 31 and 45 years of age (the mean age was 37.25 years with 0.711 standard deviations). More than half (54.7%) and 77 (12.7%) of study participants were married and divorced, respectively. Nearly a quarter, 136 (22.5%) of the participants, had no formal education, and more than half, 328 (54.2%) participants, had income less than 1200 Ethiopian Birr (ETB). About two-thirds (66.6%) of respondents were from urban residents (Table 1).

**Table 1.** Socio-demographic characteristics of participants using ART services in public health facilities in the Wolaita zone south Ethiopia 2021.

Variables		Frequency	Percent (%)
Sex	Male	261	43.1
	Female	344	56.9
Age	18–31 years	182	30.1
	32–45 years	327	54.0
	46–59 years	84	13.9
	≥60 years	12	2.0
Resident	Urban	399	66.0
	Rural	206	34.0
Educational status of clients	No formal education	136	22.5
	Primary education	211	34.9
	Secondary education	188	31.1
	College/University	70	11.6
Occupational status of clients	Farmer	116	19.2
	Merchant	109	18.0
	Government employee	88	14.5
	Daily labour	118	19.5
	Private organisations	54	8.9
	Non-worker	65	10.7
	Others*	55	9.1
Marital status of respondents	Single	140	23.1
	Married	331	54.7
	Divorced	77	12.7
	Widowed	57	9.4
Ethnicity of study participants	Wolaita	479	79.2
	Silte	21	3.5
	Amhara	50	8.3
	Gurage	37	6.1
	Others**	18	3.0
The Religion of study participants	Protestants	291	48.1
	Orthodox	261	43.1
	Muslims	29	4.8
	Catholic	24	4.0
Income	<1200 ETB	328	54.2
	≥1200 ETB	277	45.8
Name of facility	Boditti HC	115	19.0
	Dubo GH	162	26.8
	Areka HC	52	8.6
	Sodo HC	221	36.5
	Bele PH	38	6.3
	Shanto HC	17	2.8

Other* = includes (Housewife & Driver) and Others** = includes (Kambata, Hadiya, Gamo, Oromo).

### Clients response to the process of service provision and resource-related issues

About two-thirds of study participants, 419 (69.3%), agreed on the availability of prescribed laboratory services in their facility. In contrast, 278 (46.0%) respondents disagreed on the availability of prescribed drugs. More than half of the study participants (50.9%) did not feel happy about the facility’s toilet cleanliness. About one-fourth of respondents did not agree on the technical competency of service providers and the availability of separate adherence units in the facility (Table 2).

**Table 2.** Client response on the process of service provision and resource-related issues in Wolaita zone health facilities, 2021.

Variables		Frequency	Per cent (%)
Availability of prescribed laboratory services	Yes	419	69.3
	No	186	30.7
Availability prescribed drugs	Yes	327	54.0
	No	278	46.0
Cleanliness of toilet in the facility	Yes	297	49.1
	No	308	50.9
Cleanliness of facility	Yes	266	44.0
	No	339	56.0
The comfort of the waiting area	Yes	409	67.6
	No	196	32.4
Separate adherence unit in the facility	Yes	448	74.0
	No	157	26.0
Technical competence of service providers	Yes	450	74.4
	No	155	25.6
Distance home to facility	<30 minutes	209	34.5
	30 min − 1 hour	246	40.7
	1–2 hours	113	18.6
	>2 hours	37	6.1

### Client satisfaction with ART services received from public health facilities

The overall client satisfaction with ART service provision was 70.70%. Accordingly, about half of the clients agreed (55.5%) with the standard waiting time to get services (mean ± standard deviation) (4.12 ± 0.710) and half of the participants (50.2%) agreed with the willingness to service providers to respond to complaints. Half (50.6%) of ART clients strongly agree that staff treat all patients fairly and equally. On the other hand, about 47.4% of the clients strongly agreed that service providers ensure their medication was taken as prescribed (Table 3).

**Table 3.** ART clients’ response to ART services from the public health facilities of Wolaita Zone south Ethiopia, 2021.

Variables	Strongly Disagree No (%)	Disagree No (%)	Uncertain No (%)	Agree No (%)	Strongly agree No (%)	Mean (SD)
Ensuring clients take their medication as prescribed	0 (0)	3 (0.5)	92 (15.2)	223 (36.9)	287 (47.4)	4.31 (± 0.741)
ART clients care in the clinics	0 (0)	12 (2.0)	160 (26.4)	265 (43.8)	168 (27.8)	3.97 (± 0.789)
Staff treats all patients fairly	2 (0.3)	9 (1.5)	55 (9.1)	233 (38.5)	306 (50.6)	4.38 (± 0.739)
Client response on privacy	2 (0.3)	9 (1.5)	55 (9.1)	233 (38.5)	306 (50.6)	4.25 (± 0.623)
Client response on confidentiality	2 (0.3)	9 (1.5)	55 (9.1)	233 (38.5)	306 (50.6)	3.96 (± 0.726)
Standard waiting time to get services	1 (0.2)	12 (2.0)	78 (12.9)	336 (55.5)	178 (29.4)	4.12 (± 0.710)
Politeness of service providers	2 (0.3)	11 (1.8)	61 (10.1)	317 (52.4)	214 (35.4)	4.21 (± 0.718)
Providers treat opportunistic infections	0 (0)	0 (0)	39 (6.4)	327 (54.0)	239 (39.5)	4.33 (± 0.592)
Providers interaction or communication	0 (0)	4 (0.7)	47 (7.8)	341 (56.4)	213 (35.2)	4.26 (± 0.623)
Explanation or information guidance	32 (5.3)	62 (10.2)	76 (12.6)	253 (41.8)	182 (30.1)	3.81 (± 1.131)
Willingness of providers to respond to complaints	0 (0)	2 (0.3)	135 (22.3)	304 (50.2)	164 (27.1)	4.04 (± 0.712)
Listening carefully clients concern	1 (0.2)	15 (2.5)	174 (28.8)	222 (36.7)	193 (31.9)	3.98 (± 0.849)
Availability of providers all-time in the ART room	0 (0)	47 (7.8)	110 (18.2)	283 (46.8)	165 (27.3)	3.94 (± 0.873)
Consultation time to explain medications	1 (0.2)	10 (1.7)	121 (20.0)	271 (44.8)	202 (33.4)	4.10 (± 0.779)

### Clients’ satisfaction with antiretroviral therapy (ART) services

Out of 605 study participants, 428 (70.7%) respondents had satisfaction with the antiretroviral treatment service in the Wolaita zone. The overall satisfaction specific to each health facility ranged from 21.1% to 90.0%. Sodo Health Centre (90.0%) and Areka Health Centre (84.6%) showed the highest client satisfaction rate with antiretroviral services, while Bele Primary hospital (21.1%) and Boditti Health Centre (34.8%) showed the lowest satisfaction rate.

Male clients (77.0%), age group between 46 and 59 years (73.8%), wealth index less than 1200 ETB (76.5%), urban clients (74.2%), higher education or college/university (75.8%), employed clients (88.7%), widowed (80.7%) and distance to reach health facility within 30 minutes to 1 hour (72.4%) showed the highest satisfaction rate. While among clients’ responses, the highest satisfaction was reported for clients whose response ‘yes there is’ on the availability of prescribed laboratory service (75.4%), availability of prescribed drugs (83.2%), cleanliness of toilet (75.8%), cleanliness of facility (78.2%) and comfort of waiting for the area (73.1%).

### Predictors of client satisfaction with antiretroviral treatment service

The study participants’ socio-demographic, economic, process of service provision and resource-related variables were first analysed in bivariate analysis using a binary logistic regression model to select candidate variables for multivariable analysis. Male study participants were more satisfied than females [AOR = 1.91, 95 CI (1.10, 3.29)], which had a statistically significant association with client satisfaction with ART services. Employed study participants were more satisfied with ART services than not employed [AOR = 13.04, 95 CI (4.34, 39.22)] and associated with client satisfaction with ART services (Table 4).

**Table 4.** Multivariable analysis of predictors to assess associated factors of clients’ satisfaction with ART services in Wolaita Zone, 2021.

Variables		ART Service Satisfaction		COR (95% CI)	AOR (95% CI)	*P*-value
		SatisfiedN(%)	Not Satisfied N(%)			
Sex	Male	201 (77.0)	60(23.0)	1.73(1.20,2.49)	1.91(1.10,3.29)	0.02
	Female	227 (66.0)	117(34.0)	1	1	
Age	18–31	131 (72.0)	51(28.0)	1		
	32–45	228 (69.7)	99(30.3)	1.12(0.75,1.66)		0.59
	46–59	62 (73.8)	22(26.2)	0.91(0.51,1.64)		0.75
	≥60	7 (58.3)	5(41.7)	1.84(0.56,6.04)		0.31
Resident	Urban	296 (74.2)	103(25.8)	1.61(1.12,2.32)	1.41(0.75,2.65)	0.29
	Rural	132 (64.1)	74(35.9)	1	1	
Educational status	No formal education	103 (75.3)	33(24.3)	0.74(0.40,1.37)	0.79(0.40,1.77)	0.37
	Primary education	147 (69.7)	64(30.3)	0.64(0.34,1.19)	0.69(0.31,1.28)	0.18
	Secondary education	125 (66.5)	63(33.5)	1.00(0.51,1.96)	1.00(0.45,1.99)	0.99
	College	53 (75.7)	17(24.3)	1	1	
Occupational status	Farmer	75 (64.7)	41(35.3)	1.38(0.74,2.58)	1.68(0.61,4.68)	0.31
	Merchants	76 (69.7)	33(30.3)	1.74(0.92,3.01)	1.67(0.63,4.34)	0.30
	Employed	126 (88.7)	16(11.3)	5.96(2.92,12.18)	13.04(4.34,39.2)	0.001
	Daily labor	114 (65.9)	59(34.1)	1.46(0.82,2.62)	1.15(0.47,2.80)	0.76
	Not employed	37 (56.9)	28(43.1)	1	1	
Marital status	Single	96 (68.6)	44(31.4)	0.52(0.25,1.10)	0.39(0.12,1.24)	0.1
	Married	232 (70.1)	99(29.9)	0.56(0.28,1.13)	0.58(0.22,1.55)	0.27
	Divorced	54 (70.1)	23(29.9)	0.56(0.25,1.27)	0.61(0.21,1.79)	0.37
	Widowed	46 (80.7)	11(19.3)	1	1	1
Availability prescribed laboratory services	Yes	316(75.4)	103(24.6)	2.03(1.40, 2.93)	2.56(1.42, 4.63)	0.002
	No	112(60.2)	74(39.8)	1	1	
Availability prescribed drugs	Yes	272(83.2)	55(16.8)	3.87(2.66,5.62)	6.26(3.40,11.52)	0.001
	No	156(56.1)	122(43.9)	1	1	
Cleanliness of toilet	Yes	225(75.8)	72(24.2)	1.62(1.13,2.31)	2.83(1.56,5.14)	0.001
	No	203(65.9)	105(34.1)	1	1	
Cleanliness of facility	Yes	208(78.2)	58(21.8)	1.94(1.34,2.80)	1.33(0.77,2.28)	0.309
	No	220(64.9)	119(35.1)	1	1	
Separate adherence unit	Yes	320(71.4)	128(28.6)	1.13(0.76,1.68)		0.532
	No	108(68.8)	49(31.2)	1		
Perceived technical competency of service providers	Yes	320(71.1)	130(28.9)	1.07(0.72,1.60)		0.735
	No	108(69.7)	47(30.3)	1		

Clients who received prescribed laboratory services were 2.56 more likely to be satisfied with ART services than those who did not [AOR = 2.56; 95% CI: (1.42, 4.63)], and clients who received prescribed drugs in the facility were 6.26 more likely to be satisfied than those who did not receive the prescribed drugs [AOR = 6.26; 95% CI: 6.26 (3.40, 11.52)]—had a significant association with ART services. Also, the cleanliness of the toilet was associated with client satisfaction with ART services [AOR = 2.83; 95% CI (1.56, 5.14)].

## Discussion

This study showed that the overall client satisfaction with antiretroviral treatment services provided in public facilities was 70.7% [95% CI (66.94%, 74.34%)]. The present study result was similar to a study conducted in Hossana, Ethiopia, with overall client satisfaction of 70.10% [6]. However, the satisfaction level was higher than the study conducted in Vietnam, 42.4%, India, 61.3%, and Wollega, Ethiopian studies, 57.2% [3,4,19]. The client satisfaction measurement had significant implications for service providers and planners to identify gaps and improve the service quality of the health care system.

However, this study found the lowest satisfaction rate compared to the study conducted in Nigeria, 99.6% [18], Cameroon 91.2% [20] and Ethiopia (Tigray region 89.6% [10], Addis Ababa 85.5% [17] and Northwest Ethiopia 85.3%) [2]. This variation might be due to better diagnostic facilities, good infrastructure, and qualified and adequate health professionals in the above study areas. The other reason might be variation in the availability and achievement of implementation programmes for improving patient satisfaction in the facilities or countries. In addition to studies in Addis Ababa and Tigray hospitals, patient satisfaction was measured focused on laboratory and pharmacy services. Furthermore, hospitals with better diagnostic and medical personnel for better health care service provision might fulfill patient expectations compared to health centres.

Sex was found to have a statistically significant association with client’s satisfaction with ART services; males were 1.91 times more likely to be satisfied, which was consistent with Cameroon and Vietnam studies as it showed that males were more satisfied with ART services than females and significantly associated with ART services [19,20]. The reason might be that most of the female study participants required additional services related to their sex. The other justification could be that in-home activity highly influences females in developing countries, or they could come to the health facility after experiencing various responsibilities and duties in the household; a long waiting time might cause low satisfaction.

Employed study participants were more satisfied with ART services or 13.04 [AOR = 13.04, 95 CI (4.34, 39.22)] times more likely to be satisfied with ART services than those not employed. This finding was similar to a study conducted in Cameroon [20]. Client satisfaction was strongly associated with the availability of prescribed laboratory services in facilities; those who received such services were 2.56 times more likely to be satisfied. The findings from this study were consistent with those of studies carried out in Nigeria [1] and Ethiopia (Hossana, Tigray, Jimma, and Addis Abeba), which demonstrated strong association between the availability of laboratory services and client satisfaction with antiretroviral services [6,10,17,21]. The reason might be the availability of prescribed laboratory services in the facility where the client wants all laboratory services.

The current study also showed that the availability of prescribed drugs in the facility was significantly associated with satisfaction with ART services, and the availability of prescribed drugs was found to be 6.26 times more likely to satisfy clients. This study finding was concordant with Nigeria and Ethiopia’s studies [10,18]. The primary justification for this finding might be that clients who did not get drugs from the facility could incur additional costs to buy the drugs from private pharmacies, resulting in dissatisfaction with the overall service in the facility [10].

The facility’s toilet cleanliness had a statistically significant association with client satisfaction. The cleanliness of the toilet was 2.83 times more likely to satisfy clients. This finding was consistent with a study conducted in Tigray, Ethiopia, which showed the cleanliness of toilets in the facilities related to patient satisfaction in the ART clinics [10]. Lower satisfaction towards providing a clean toilet to the client’s response was observed in a study done in Bangladesh, which showed that satisfaction with the cleanness of the toilet was only 54% [22]. About half (51%) of study participants reported poor toilet cleanliness in the health facility, and the existing toilet was too dirty. According to the remark part of an interview of this study, due to uncomfortable/dirty toilets, clients were forced to use the open defaecation in the facility compound; this could decrease the client’s satisfaction with the facility and ART services.

### Strengths and limitations

Although this study used a large sample size and multiple variables, it lacks investigation into the clinical characteristics, such as stage of AIDS diseases and viral loads; those developed opportunistic infections that might be required add the need for service of study participants related to ART service satisfaction. Moreover, the dichotomisation of Likert scale measurements might introduce misclassification and disruption of the data’s nature.

## Conclusion

The overall client satisfaction with antiretroviral treatment service was lower than the national target (85%). Sex (male) and employment were positively associated with clients’ satisfaction with ART services among socio-demographic characteristics, or both are the determinants for client satisfaction with ART services. The availability of prescribed laboratory services, prescribed drugs, and the cleanliness of toilets in the facility were factors associated with client satisfaction with antiretroviral treatment services. Tangibility aspects of the client’s response on the cleanliness of toilets in the facility were poor, or toilets in those health facilities were dirty or unclean. Procurement and supply for the availability of laboratories and medicine require attention. Sex-sensitive programmes should be considered to improve client satisfaction. Further studies are needed, including clinical characteristics of ART clients.

## Paper context

A human immune virus is the leading cause of morbidity and mortality worldwide, but sub-Saharan countries are highly affected. Ethiopia’s government has been working on comprehensive HIV care and treatment programmes but evaluating client satisfaction with antiretroviral treatment services is not well studied. The overall client satisfaction was lower than the national target. Ethiopia’s lack of comprehensive laboratory services and standard drugs was associated with client satisfaction. Sustained availability of laboratory services and medicine is recommended.
